# Relationship between mentalizing and teacher burnout: A cross sectional study

**DOI:** 10.1371/journal.pone.0279535

**Published:** 2023-01-13

**Authors:** Teodora Safiye, Branimir Vukčević, Ardea Milidrag, Jakša Dubljanin, Azra Gutić Cikotić, Draško Dubljanin, Maja Lačković, Ivana Rodić, Milica Nikolić, Goran Čolaković, Tatjana Mladenović, Medo Gutić

**Affiliations:** 1 Faculty of Medical Sciences, University of Kragujevac, Kragujevac, Serbia; 2 High School of Culinary Arts and Tourism with Dormitory, Vrnjačka Banja, Serbia; 3 Department of Cardiology, University Clinical Hospital Center Zemun, Belgrade, Serbia; 4 General Hospital Berane, Berane, Montenegro; 5 Department of Pulmonology, University Clinical Hospital Center Zvezdara, Belgrade, Serbia; 6 Psychiatry Clinic, University Clinical Center of Serbia, Belgrade, Serbia; 7 Faculty of Medicine, University of Belgrade, Belgrade, Serbia; 8 Ministry of Health of the Republic of Serbia, Inspection sector, Belgrade, Serbia; 9 Institute for Urgent Medical Care, Belgrade, Serbia; 10 Community Health Center Novi Beograd, Belgrade, Serbia; 11 Public Health Institution Health Center "Dr Branko Zogovic", Plav, Montenegro; Panimalar Medical College Hospital & Research Institute, INDIA

## Abstract

**Background:**

Teaching is considered a high-risk profession due to the high impact of occupational risk factors which can endanger educators’ mental health and lead to burnout syndrome. This study aimed to examine whether the capacity for mentalizing in teachers explains the degree of their burnout syndrome. The expectation was that a low capacity for mentalizing increases the degree of burnout.

**Methods:**

A cross-sectional study was conducted on a sample of 823 teachers. The Maslach Burnout Inventory-Educators Survey was used to examine the burnout syndrome. The capacity for mentalizing was examined using hypomentalizing and hypermentalizing scales from the Reflective Functioning Questionnaire.

**Results:**

The expectation that a low capacity for mentalizing increases teachers’ burnout confirms the finding that hypomentalizing is a positive predictor of their emotional exhaustion as a dimension of burnout (*ß* = 0.09; *p* < 0.01). Unexpectedly, hypomentalizing proved to be a positive predictor of personal accomplishment (*ß* = 0.09; *p* < 0.05), which indicates that with a lower capacity for mentalizing, teachers experience greater personal accomplishment. Also, hypermantalizing was a negative predictor of emotional exhaustion (*ß* = -0.17; *p* < 0.01) and depersonalization (*ß* = -0.31; *p* < 0.01), and a positive predictor of personal accomplishment (*ß* = 0.30; *p* < 0.01). The findings showed that with higher socioeconomic status, with marriage and having children, the burnout of teachers is lower, as expected.

**Conclusions:**

Capacity for mentalizing and burnout syndrome in teachers are interrelated phenomena. With a good capacity for mentalizing, emotional exhaustion and burnout in teachers are reduced. Knowledge and skills that enable a good capacity for mentalizing should be included in educational and teacher training programs.

## Introduction

Teaching is considered a high-risk profession due to the high impact of occupational risk factors on educators’ mental health [1]. Research conducted in the last twenty years shows that the teaching profession is very stressful and that primary and secondary school teachers are at risk of getting burnout symptoms [2]. Burnout is understood as a special form of long-term occupational stress, which has negative consequences on job satisfaction, professional efficiency, and the mental health of workers. Evidence from the literature indicate that the most important socio-demographic characteristics associated with burnout are gender, age, level of education and marital status [3]. Even though there are conflicting results in literature, research generally evidences how female workers with lower education status and less working experience are the most vulnerable to burnout [3]. Concerning to marital status, those who are unmarried seem to be more prone to burnout compared with those who are married [4]. When it comes to age, previous research has found that there was a significant negative correlation between age and burnout syndrome [3]. The study of burnout among primary and secondary school teachers is of particular importance, both because of the teachers and students themselves, and because of the great social importance of these professions [5]. Burnout among teachers has at least four sources: the first is called bad behavior of students, and includes lack of motivation in students and a negative attitude towards school obligations, indiscipline of students in class, disruption of classes, physical, psychological and/or emotive workplace violence by students or pupils etc. [6]; the second source was named as poor working environment conditions, which include lack of teaching aids, too many students in the class, low incomes, inability to advance, etc.; the third is called lack of time or too much work, and includes lack of time for preparation of classes, for assessment, for individual work with students, for personal development, etc.; the fourth source is named as a bad school ethos, which includes bad interpersonal relations with colleagues, bad relations with the school management, bad way of managing the school, etc. [5, 7]. Initially, burnout was defined as psychosocial stress that occurs primarily in professions that involve direct contact with other people, and it is believed that personal competencies in communication and cooperation can prevent burnout [4]. Psychosocial factors are the class of occupational risk agents that has recently attracted the attention of researchers. Due to the nature of the teaching profession as a human service profession with the basic task of educating children, psychosocial factors of work are of particularly high relevance [8–10]. Teachers have to deal with various work-related stressors and psychosocial risk factors, including emotional demands in the relationships with parents and students, high workload, time pressure, emotional labor and others. Based on the specific job demands, it is considered that the teaching profession is associated with a higher vulnerability to mental distress than many other occupations [3, 9, 10]. Harris et al. [11] show that the socio-affective skills of teachers are a major prevention factor in their burnout. Schwarzer et al. [12] show that global distress in teachers is associated with a weaker capacity for mentalizing as an aspect of their social skills. Dexter and Wall [13] show that a good reflective function of the self, which implies the capacity for mentalizing, increases teachers’ experience of their efficiency and thus reduces their burnout level.

### Burnout syndrome

Burnout is conceptualized as a syndrome consisting of three dimensions: a) emotional exhaustion; b) depersonalization or cynicism; c) reduced experience of personal accomplishment. Emotional or mental exhaustion is a core element of burnout, and it refers to a lack of energy and chronic fatigue caused by doing work. Depersonalization or cynicism in the teaching profession manifests itself in the form of negative attitudes toward students, as well as in the form of reduced capacity to be sensitive and responsive to the needs of students. Decreased experience of personal accomplishment refers to the negative evaluation of one’s work and the perception that work is not done well [4, 14, 15].

### Mentalizing

Mentalizing is a form of imaginative mental activity that consists of interpreting perceived human behavior based on intentional mental states such as needs, desires, feelings, beliefs, goals, purposes, and reasons. Mentalizing is a process that enables individuals to correctly understand their own and other people’s behavior in interpersonal relationships, as well as to regulate their own emotions and impulses well. In direct contact with another person, the basic mental actions that an individual performs when mentalizing are making assumptions about the mental states that determine behavior and checking them. Then the individual is aware that intentional mental states cannot be seen with the naked eye. During mentalizing, an individual has a not-knowing stance about intentional mental states and a sincere curiosity that helps him discover them in cooperation with another person [16–18]. Low capacity for mentalizing has been found in patients with borderline personality disorder, but other mental disorders also include difficulties in mentalizing [18, 19]. Also, in the non-clinical population, forms of impaired mentalizing capacity were examined. Two such forms were investigated in these studies: hypomentalizing and hypermentalizing. Hypomentalizing refers to the lack or absence of consideration of the phenomena of mental life that determine behavior, which takes place through the set of assumptions and their verification in interpersonal interaction. Hypomentalizing may be the result of a lack of faith in one’s own ability to know the mental world, or as a result of erroneous beliefs that behavior is determined by external forces rather than mental states. Hypermentalizing refers to making too many assumptions about intentional mental states, some of which are uncritically accepted as true, even though they are not true. It manifests itself as excessive certainty in the accuracy of one’s own beliefs about the nature of mental states that underlie one’s behavior [17, 20].

### The objective and research hypotheses

This paper examines the relationship between mentalizing capacity and burnout syndrome in primary and secondary school teachers. The main aim was to examine whether the capacity for mentalizing can explain the degree of burnout. The following hypotheses have been set: a) significant positive correlations are expected between low capacity for mentalizing and emotional exhaustion, depersonalization (cynicism), and significant negative correlation between low capacity for mentalizing and the dimension of personal accomplishment; b) it was expected that a low capacity for mentalizing would be a positive predictor of the dimensions of burnout—emotional exhaustion and depersonalization, and a negative predictor of personal accomplishment. The following characteristics were also examined as control variables: gender, age, marital status, number of children, socioeconomic status. The choice of control variables is consistent with previous research [2, 20, 21]. To the best of our knowledge, this paper is the first in the world to examine the role of mentalizing in explaining burnout syndrome in a teacher sample.

## Methods

### Study design, sample and procedures

A cross-sectional study design was adopted. The inclusion criteria for the study sample were teachers employed in primary or secondary schools in Serbia. The required sample size was calculated using Raosoft Sample Size Calculator (http://www.raosoft.com/samplesize.html, accessed on: 1st September 2021). According to the assumption of a margin error of 5%, a confidence level of 95%, and a population size of 20.000, a sample of 377 respondents was calculated. Since the research was conducted during the COVID-19 pandemic, in order to minimize social interactions, data were collected online using the Google Forms platform from October 2021 to December 2021 in Serbia. The link to the questionnaire was sent to school principals throughout Serbia with a request to forward it to their employed teachers. The objectives of the research were explained to potential participants at the very beginning of the questionnaire in Serbian. Participation in the research was voluntary and with informed consent, and the respondents were guaranteed the confidentiality and anonymity of the obtained data.

### Ethical considerations

This research is a part of a larger self-financing project that examined burnout syndrome and the mental health of workers during the COVID-19 pandemic in Serbia, led by the first author of this paper. This study was approved by the Institutional Review Board of the University of Belgrade, Faculty of Philosophy, Department of Psychology, Serbia (Approval number: #2021–58). The procedures of this study were following the provisions of the Declaration of Helsinki on medical research involving human subjects [22]. Written informed consent was obtained from each participant prior to recruitment into the study and each participant was informed that he/she had the right to withdraw from the study at any time without any adverse consequences. Names of the participants were not revealed in the study report and all information gathered from the study participants was protected, only the research team had access.

### Outcome measures

The Maslach Burnout Inventory-Educators Survey (MBI-ES) [14] was used to measure burnout syndrome. It consists of 22 questions that are answered by assessing the frequency of each claim. A seven-point Likert scale measurement is used, where 0 means that the statement never happens, and 6 means that it happens every day. It consists of three scales: emotional exhaustion, depersonalization, and personal accomplishment. This questionnaire does not have a unique score, but the results of the three mentioned scales are presented and interpreted following the achieved results. Emotional exhaustion includes items related to the experiences of emotional overload and exhaustion from work (e.g., "I feel emotionally drained from my work"). Depersonalization includes items related to insensitive and impersonal responses to students (e.g., "I feel that I treat some students as if they are impersonal objects"). Personal Accomplishment includes items related to a sense of competence and success at work (e.g., "I can easily understand how my students feel"). The Emotional exhaustion score was calculated by adding scores from questions 1, 2, 3, 6, 8, 13, 14, 16, and 20, and the total result on this subscale could range from 0 to 56. The Depersonalization score was calculated by adding scores from questions 5, 10, 11, 15, and 22, and could range from 0 to 30. The Personal Accomplishment score was calculated by adding scores from questions 4, 7, 9, 12, 17, 18, 19, and 21, and could range from 0 to 48 [14]. A high score on the Emotional exhaustion (EE) and Depersonalization (DP) subscales are directly proportional to the degree of burnout, while the score on the Personal Accomplishment (PA) subscale is inversely proportional, ie. the greater the sense of personal accomplishment, the lower the level of burnout [14]. The analysis for internal consistency of the Serbian version of the MBI-ES questionnaire showed that Cronbach’s alpha coefficient for the emotional exhaustion subscale was 0.93, while alpha coefficients for depersonalization and personal accomplishment subscales were 0.61 and 0.77, respectively [23]. The reliability of the MBI-ES scale is very good; the Cronbach’s alpha coefficients on our sample for the subscales of emotional exhaustion, depersonalization, and personal accomplishment are 0.93, 0.75, 0.79, respectively.

The capacity for mentalizing was examined using hypomentalizing and hypermentalizing scales from the Reflective Functioning Questionnaire of 8 items (RFQ-8) [17, 24]. RFQ-8 consists of two subscales: 1) a subscale of certainty in one’s assessment of mental states (RFQ-c), where high scores indicate the phenomenon of hypermentalizing, and 2) a subscale of uncertainty in one’s own ability to assess one’s own and others’ mental states (RFQ-u), which refers to the phenomenon of hypomentalizing. The RFQ-c subscale examines the degree to which a person is convinced that he or she can accurately assess his own and others’ mental states, and an example of an item is how much a person disagrees with statements such as "Human thoughts are a mystery to me." High scores on RFQ-c represent hypermentalizing. The RFQ-u subscale measures an individual’s insecurity in their own ability to assess their own and others’ mental states. It assesses the degree to which a person agrees with statements such as "Sometimes I do things without actually knowing why." High scores represent hypomentalizing, and lower scores represent optimal mentalizing [25, 26]. The answers are evaluated on a seven-point Likert scale from 1–Strongly disagree, to 7–Strongly agree. The results achieved by respondents on both subscales of RFQ-8 can range from 0 to 3. RFQ has shown good reliability in previous studies, Cronbach’s alpha coefficient was 0.70 or more [20, 25–27]. The analysis for internal consistency of the Serbian version of the RFQ-8 questionnaire showed a Cronbach’s alpha coefficient of 0.82 for the hypermentalizing (RFQ-c) subscale and 0.70 for the hypomentalizing (RFQ-u) subscale [28].

A special questionnaire was created for the assessment of sociodemographic variables for this research. Based on previous literature on burnout syndrome [2, 20], as control variables in this study were included gender (male = 1, female = 2), age, socioeconomic status (from 1 = very poor to 5 = excellent), marital status (married = 1, single = 2), and number of children (no children = 1, one child = 2, two or more children = 3).

### Statistical analysis

To describe the instruments used in the survey, mean values, standard deviations, minimum, maximum, skewness, and kurtosis were used as measures of descriptive statistics. To check the reliability of these scales, Cronbach’s alpha coefficient was used as a measure of internal consistency. Correlation coefficients and tests of their significance were used to describe the relationships between variables. Multiple hierarchical regression analysis was used to determine whether the dimensions of mentalizing are significant predictors of each of these burnout dimensions. Statistical analysis was performed using SPSS Statistics software (IBM SPSS Statistics for Windows, Version 22.0, Armonk, NY, USA).

## Results

### Participant characteristics

A total number of 831 teachers were examined. The dataset contained 8 multivariate outliers, which were identified using the Mahalanobis distance and were eliminated due to a likelihood of occurrence of *p* < 0.001 [29]. The final sample included 823 respondents who met all the criteria for inclusion in the study. 758 female and 65 male respondents participated in the study. The mean age of respondents was 43.90 ± 9.27 years (age range of 23–64 years). Out of a total of 823 respondents, 589 (71.6%) were married, and 234 (28.4%) were single. 230 (27.9%) respondents did not have children, 193 (23.5%) respondents had one child, and 400 (48.6%) stated that they had two or more children. On a scale from 1 to 5, the largest number of respondents, 476 (57.8%) rated their socioeconomic status as good (score 3), 167 (20.3%) as very good, 24 (2.9%) as excellent, 123 (14.9%) as poor, and 33 (4%) as very poor.

### Measures of descriptive statistics of the investigated variables

Table 1 shows that the values of kurtosis are between -3 and 3, while the values of skewness are between 1 and -1, except for the depersonalization and hypomentalizing scales where they are above 1, which indicates that most respondents have depersonalization and hypomentalizing values below-average values of this sample. Almost all instruments used in this study had satisfactory or good reliability (which was expressed as Cronbach’s alpha coefficient of internal consistency), as was expected.

**Table 1:** Descriptive statistics of used scales.

Scale	Min	Max	Mean	SD	Skew	Kurt	α
Depersonalization (DP)	0	27	6.05	5.43	1.12	1.07	0.75
Emotional exhaustion (EE)	0	54	30.29	12.93	-0.31	-0.71	0.93
Personal accomplishment (PA)	0	48	35.32	6.35	-0.59	1.11	0.79
Hypermentalizing (RFQ–c)	.00	3.00	1.19	0.82	0.29	-.92	0.80
Hypomentalizing (RFQ–u)	.00	2.50	.43	0.50	1.40	1.52	0.67

### Measures of descriptive statistics of burnout dimensions by sociodemographic characteristics

In the following, we will talk in more detail about the results of descriptive statistics (means and standard deviations) of three burnout dimensions according to different categories of sociodemographic variables.

When it comes to gender, in men (n = 65) the mean of emotional exhaustion was 27.43 (SD = 14.36), depersonalization 7.91 (SD = 6.53), and personal accomplishment 34.60 (SD = 6.56). In women (n = 758) the mean of emotional exhaustion was 30.53 (SD = 12.78), depersonalization 5.89 (SD = 5.30), and personal accomplishment 35.39 (SD = 6.34).

When it comes to length of service, respondents with up to 9 years of service (n = 201) had a mean value of emotional exhaustion of 27.95 (SD = 13.86), depersonalization of 6.34 (SD = 6.02), and personal accomplishment of 34.91 (SD = 6.81). Respondents with 10 to 19 years of work experience (n = 280) had a mean value of emotional exhaustion of 30.40 (SD = 12.69), depersonalization of 6.23 (SD = 5.39), and personal accomplishment of 35.30 (SD = 6.30). Respondents with 20 to 29 years of work experience (n = 241) had a mean value of emotional exhaustion of 31.54 (SD = 12.25), depersonalization of 5.84 (SD = 4.89), and personal accomplishment of 35.42 (SD = 6.19). Respondents with 30 to 40 years of work experience (n = 101) had a mean value of emotional exhaustion of 31.63 (SD = 12.80), depersonalization of 5.45 (SD = 5.50), and personal accomplishment of 35.98 (SD = 5.97).

Concerning to marital status, single respondents (n = 234) had a mean value of emotional exhaustion of 32.24 (SD = 12.81), depersonalization of 7.15 (SD = 6.01), and personal accomplishment of 34.52 (SD = 6.66). Married respondents (n = 589) had a mean value of emotional exhaustion of 29.51 (SD = 12.91), depersonalization of 5.61 (SD = 5.12), and personal accomplishment of 35.64 (SD = 6.20).

When it comes to the number of children, among respondents without children (n = 230) the mean of emotional exhaustion was 31.13 (SD = 12.55), depersonalization 7.34 (SD = 6.04), and personal accomplishment 34.14 (SD = 6.70). In respondents with one child (n = 193), the mean of emotional exhaustion was 30.95 (SD = 13.73), depersonalization 5.94 (SD = 5.59), and personal accomplishment 36.03 (SD = 5.66). In respondents with two or more children (n = 400), the mean of emotional exhaustion was 29.48 (SD = 12.73), depersonalization 5.35 (SD = 4.82), and personal accomplishment 35.67 (SD = 6.39).

Among respondents who assess their socioeconomic status as very poor (n = 33), the mean of emotional exhaustion was 38.73 (SD = 15.04), depersonalization 8.85 (SD = 7.32), and personal accomplishment 32.85 (SD = 8.76). Among the respondents who assess their socioeconomic status as poor (n = 123), the mean of emotional exhaustion was 35.91 (SD = 11.25), depersonalization 7.54 (SD = 5.41), and personal accomplishment 33.33 (SD = 5.72). Among respondents who assess their socioeconomic status as good (n = 476), the mean of emotional exhaustion was 30.20 (SD = 11.89), depersonalization 5.89 (SD = 5.23), and personal accomplishment 35.19 (SD = 6.34). Among the respondents who assess their socioeconomic status as very good (n = 167), the mean of emotional exhaustion was 26.21 (SD = 13.54), depersonalization 5.06 (SD = 5.12), and personal accomplishment 37.06 (SD = 5.64). Among the respondents who assess their socioeconomic status as excellent (n = 24), the mean of emotional exhaustion was 19.88 (SD = 15.58), depersonalization 4.42 (SD = 5.90), and personal accomplishment 39.42 (SD = 5.71).

When it comes to the age of teachers, those up to 29 years (n = 65) had a mean value of emotional exhaustion of 28.48 (SD = 14.09), depersonalization of 7.37 (SD = 6.79), and personal accomplishment of 34.46 (SD = 6.96). Respondents aged 30 to 39 (n = 188) had a mean value of emotional exhaustion of 28.63 (SD = 12.81), depersonalization of 5.91 (SD = 5.49), and personal accomplishment of 35.23 (SD = 6.44). Respondents aged 40 to 49 (n = 334) had a mean value of emotional exhaustion of 30.83 (SD = 12.79), depersonalization of 6.33 (SD = 5.39), and personal accomplishment of 35.50 (SD = 5.78). Respondents aged 50 and over (n = 236) had a mean value of emotional exhaustion of 31.33 (SD = 12.80), depersonalization of 5.39 (SD = 4.92), and personal accomplishment of 35.39 (SD = 6.89).

### The relationships among investigated variables

Table 2 shows that emotional exhaustion was statistically significantly positively correlated with hypomentalizing (r = 0.22, p < 0.01). The result indicates that with a weaker capacity for mentalizing, respondents also experience greater emotional exhaustion from work. Emotional exhaustion is negatively associated with hypermentalizing (r = -0.25, p < 0.01), which indicates that with increasing hypermentalizing, the experience of emotional exhaustion at work decreases. Emotional exhaustion was statistically significantly negatively associated with self-assessment of the degree of socioeconomic status (r = -0.28, p < 0.01). The higher the socioeconomic status of one’s socioeconomic status, the less the experience of emotional exhaustion from work.

**Table 2:** Correlations between variables.

	Gen.	Age	MS	NC	SES	RFQ–c	RFQ–u	EE	DP
Age	**.15** ^**^								
MS	**-.11** ^**^	**-.15** ^**^							
NC	**.19** ^**^	**.37** ^**^	**-.52** ^**^						
SES	.03	**-.08** ^*^	**-.13** ^**^	-.01					
RFQ–c	.02	-.00	-.02	.04	**.08** ^*^				
RFQ–u	.01	-.01	-.02	-.02	**-.07** ^*^	**-.60** ^**^			
EE	.06	**.10** ^**^	**.09** ^**^	-.05	**-.28** ^**^	**-.25** ^**^	**.22** ^**^		
DP	**-.10** ^**^	**-.07** ^*^	**.12** ^**^	**-.15** ^**^	**-.17** ^**^	**-.36** ^**^	**.25** ^**^	**.53** ^**^	
PA	.03	.03	**-.08** ^*^	**.09** ^**^	**.21** ^**^	**.26** ^**^	**-.10** ^**^	**-.26** ^**^	**-.45** ^**^

Gen.–gender; MS–marital status; NC–number of children; SES–socioeconomic status; RFQ–c–hypermentalizing; RFQ–u–hypomentalizing; EE–emotional exhaustion; DP–depersonalization; PA–personal accomplishment

** *p* < 0.01

* *p* < 0.05; statistical significant correlations are bolded.

Depersonalization was statistically significantly negatively correlated with hypermentalizing (r = -0.36, *p* < 0 .01). The finding indicates that the higher the degree of hypermentalizing in the respondents, the lower the degree of self-assessed depersonalization. Depersonalization was significantly statistically positively correlated with hypomentalizing (r = 0.25, *p* < 0.01). Along with the increase in the degree of hypomentalizing, the degree of depersonalization increased. Among other things, with the observation of higher socioeconomic status, respondents estimate that they have a lower degree of depersonalization (r = -0.17, *p* < 0.01). The findings suggest that the experience of depersonalization decreases with the increase in the number of children (r = -0.15, *p* < 0.01), as well as with the age of the respondents (r = -0.07, *p* < 0.05), and also, that with females the experience of depersonalization is lower (r = -0.10, *p* < 0.01).

The experience of personal accomplishment at work increases with increasing hypermentalizing, or decreases with decreasing hypermentalizing (r = 0.26, *p* < 0.01). With the increase of hypomentalizing, the experience of personal accomplishment at work decreases (r = -0.10, *p* < 0.01). With a higher assessment of one’s socioeconomic status, at the same time, there is the experience of greater personal accomplishment (r = 0.21, *p* < 0.01). The findings suggest that personal accomplishment at work is higher with marital than single status (r = -0.15, *p* < 0.05); as well as with a higher number of children (r = 0.09, *p* < 0.01).

Table 3 shows the results of all regression analyses. In each analysis, the variance inflation factor (VIF) had a value below 5, which indicates that there were no severe problems of multicollinearity. The value of the Durbin-Watson coefficient in the first analysis was 1.96, in the second 1.85, and the third 2.01, which indicates that there were no severe autocorrelation problems in the models [29].

**Table 3:** Hierarchical linear regression analysis of the relationship among teachers’ burnout dimensions and mentalizing.

	Outcome Variable: Emotional Exhaustion						Outcome Variable: Depersonalization						Outcome Variable: Personal Accomplishment					
	Control Variables			Control Variables and Predictors			Control Variables			Control Variables and Predictors			Control Variables			Control Variables and Predictors		
	*ß*	*T*	VIF	*ß*	*t*	VIF	*ß*	*t*	VIF	*ß*	*t*	VIF	*ß*	*t*	VIF	*ß*	*t*	VIF
Gen.	**.08** *	2.38	1.05	**.08** *	2.48	1.05	-.06	-1.84	1.05	-.06	-1.85	1.05	.00	.17	1.05	-.00	-.02	1.05
Age	**.10** **	3.01	1.18	**.10** **	3.03	1.18	-.03	-.82	1.18	-.03	-1.05	1.18	.01	.44	1.18	.02	.66	1.18
MS	.03	.80	1.41	.04	1.07	1.42	.03	.88	1.41	.04	1.16	1.42	-.00	-.06	1.41	.00	-.00	1.42
NC	**-.10** *	-2.42	1.59	**-.08** *	-2.08	1.60	**-.11** **	-2.61	1.59	**-.09** *	-2.23	1.60	**.08** *	2.02	1.59	.07	1.81	1.60
SES	**-.27** **	-8.29	1.03	**-.25** **	-7.86	1.04	**-.17** **	-4.92	1.03	**-.14** **	-4.32	1.04	**.21** **	6.30	1.03	**.20** **	6.00	1.04
RFQ–c				**-.17** **	-4.38	1.57				**-.31** **	-7.76	1.57				**.30** **	7.45	1.57
RFQ–u				**.09** **	2.42	1.57				.05	1.42	1.57				**.09** *	2.39	1.57
R^2^	.10			0.16			.06			.17			0.5			0.12		
adj. R^2^	.10			0.15			.05			.17			0.5			0.11		
F Ch.	**19.27** **			**29.54** **			**10.35** **			**59.10** **			**9.69** **			**31.15** **		

Gen.–gender; MS–marital status; NC–number of children; SES–socioeconomic status; RFQ–c–hypermentalizing; RFQ–u–hypomentalizing

** *p* < 0.01

* *p* < 0.05; statistical significant correlations are bolded.

The emotional exhaustion regression model that included control and predictor variables explained 5% more variance of emotional exhaustion than the model with control variables alone. Hypermentalizing was a significant negative predictor of emotional exhaustion (*ß* = -0.17; *p* < 0.01), which means that with the increase in the degree of experienced hypermentalizing, the degree of experienced emotional exhaustion decreased. Hypomentalizing was a significant positive predictor of emotional exhaustion (*ß* = 0.09; *p* < 0.01), which shows that with the increase in hypomentalizing, emotional exhaustion also increased. When it comes to controlling variables, emotional exhaustion is mostly explained by socioeconomic status (*ß* = -0.25; *p* < 0.01). The higher the socioeconomic status of the respondents, the lower the experience of emotional exhaustion at work. Also, with a higher number of children, the experience of emotional exhaustion at work among the respondents was lower (*ß* = -0.08; *p* < 0.05), while with older age the experience of exhaustion was higher (*ß* = 0.10; *p* < 0.01).

The regression model of depersonalization, which included control and predictor variables, explained 12% more variance of depersonalization than the model with control variables alone. Hypermentalizing was a significant negative predictor of depersonalization (*ß* = -0.31; *p* < 0.01), which means that together with the increase in the degree of experienced hypermentalizing, the degree of experienced depersonalization decreased. With the assessment of higher socioeconomic status, the respondents also had the experience of less depersonalization at work (*ß* = -0.14; *p* < 0.01); also, with a higher number of children, respondents had less experience of depersonalization at work (*ß* = -0.09; *p* < 0.05).

The regression model of personal accomplishment at work, which included control and predictor variables, explained 6% more variance of personal accomplishment at work than the model, which included only control variables. Hypermentalizing was a significant positive predictor of personal accomplishment (*ß* = 0.30; *p* < 0.01), which means that along with the increase in the degree of hypermentalizing experienced, the degree of personal accomplishment at work also increased. Hypomentalizing was a positive predictor of personal accomplishment (*ß* = 0.09; *p* < 0.05), which means that with the weakening of capacity for mentalizing in the respondents, the experience of personal accomplishment was higher. The coefficient of partial correlation between hypomentalizing and personal accomplishment was positive and was 0.80 (*p* < 0.05). Socioeconomic status also explains part of the variance of personal accomplishment at work as a significant positive predictor (*ß* = 0.20; *p* < 0.01). With the experience of higher socioeconomic status among the respondents, the experience of personal accomplishmentat work was also higher.

## Discussion

The main aim of this paper was to examine whether the capacity for mentalizing of teachers can explain the degree of their burnout. It was expected that the low capacity for mentalizing of teachers would explain the higher degree of their burnout. Emotional intelligence and capacity for mentalizing are “conceptual cousins” because both constructs pertain to identifying emotions in oneself as well as in other individuals, more precisely, mentalizing is the skill of understanding the emotions of others and improving relationships with others [30, 31]. Both emotional intelligence and mentalizing were found to be predictive of mental health, the quality of social relationships, and wellbeing [31], and emotional intelligence is negatively correlated with burnout [1], as well as good capacity for mentalization [28].

Two phenomena representing low capacity for mentalizing were examined: hypomentalizing, and hypermentalizing. The confirmed expectation is that the increase in hypomentalizing, ie. weakening the mentalizing capacity of teachers, increases the level of their emotional exhaustion at work, which is consistent with previous research [11, 13, 20]. Good mentalizing implies that during direct communication, empathy, active listening and authentic curiosity about mental states are expressed, both one’s own and the interlocutors. Instead of discovering objective facts about the reasons for behavior through open communication with others, hypomentalizers usually judge intentional mental states by "guessing", sometimes referring to general laws or their previous experience, which leads to wrong conclusions. With the weakness of mentalizing capacity, the ability of teachers to understand their own and other people’s behavior at work decreases, which leads to interpersonal misunderstandings, conflicts, professional frustrations, and dissatisfaction with work. This is also in line with previous findings that a good capacity for mentalizing is a protective factor for mental health [12, 16, 19]. However, the finding that indicates that hypomentalizing occurs together with the experience of personal accomplishment at work is unexpected. It can be assumed that teachers who are prone to hypomentalizing, tend to see their work mainly as giving *ex-cathedra* lectures and see themselves as effective transmitters of knowledge, neglecting advisory work with students and colleagues in which to reveal their own and others’ intentional mental states. This is a hypothesis that requires new research.

The findings showed that hypermentalizing reduces emotional exhaustion and depersonalization, and increases the experience of personal accomplishment at work. Unexpectedly, these findings indicate that hypermentalizing has a role in preventing burnout. Unlike a hypomentalizer who generally deals poorly with issues of mental life, a hypermentalizer is a person who usually does this a lot, but very poorly. The hypermentalizer builds extensive theories about his own and other people’s mental states that are not based on checking assumptions and facts that are obtained in open conversation with the interlocutor. Hypermentalizing includes, among other things, avoiding direct communication in which personal theories of mental states are put to the test [16, 19, 20]. The hypermentalizing scale used is mostly related to the degree of confidence in the infallibility of one’s assessment of one’s and others’ mental states [16, 17]. Taking into account the above, the mentioned finding in this paper shows that teachers who are inclined to firmly believe in the accuracy of their own assessments of their own and others’ mental states are also inclined to believe that they do their job efficiently. People who are hypermentalized when interpreting their own and other people’s behavior during communication believe that they do not make mistakes when setting their theories about their own and others’ intentional mental states, so they do not spend their time checking their assumptions, avoiding open and two-way communication with other persons that may produce doubt about one’s infallibility, experience frustration, or conflict with those persons [16, 19]. This finding indicates that teachers who tend to hypermentalize, doing so in the context of their work, also avoid checking their assumptions about intentional mental states through honest communication with colleagues and students, thus avoiding frustrations and difficulties, and reducing their experience of emotional exhaustion and depersonalization, and the experience of personal accomplishment grows. However, they remain deprived of a good knowledge of the intentional mental states of their own, as well as colleagues and students, which is an aspect of hypermentalizing, as already mentioned.

The findings indicated that good socioeconomic status, being married and having children, means less burnout at work for teachers, which is following previous research which showed that workers who have marriage and children, as well as a good socioeconomic position in society, find their work less stressful, which prevents burnout [2, 3, 5, 7, 12].

### Practical implications

Bearing in mind not only that the mental health of teachers is important, but also that the teaching profession is very important for children’s development and the future of society [5], two important implications of this research should be pointed out. Teachers should be provided with training programs that develop their capacity for mentalizing. Other research can be conducted to find out which knowledge and skills are most important for strengthening the mentalizing capacity of teachers in the context of their work, and following the findings of such research to modify existing teacher training programs.

### Study limitations and future directions

Since this is a study of correlation design, it cannot produce knowledge about cause-and-effect relationships. The results were interpreted so that following the findings of previous research, the emphasis was placed on the effects of mentalizing on burnout syndrome. However, the results may also be due to the effects of burnout on the capacity for mentalizing. A cross-sectional study cannot answer the question of the extent to which the dimensions of burnout syndrome diminish the capacity for mentalizing in teachers over time. Further longitudinal studies is needed to solve this problem. Because we used a self-reporting questionnaire in data collection, self-reporting bias may be present [32]. Also, one of the limitations could be that this study was conducted during the COVID-19 pandemic. However, there is no empirical evidence suggesting that the relationship between capacity for mentalizing and burnout dimensions varies depending on the pandemic situation. Future research could be designed to better discover and explain the relationship between hypomentalizing and hypermentalizing with different aspects of teacher work, such as teaching students or teamwork with colleagues. Such findings could be used in creating teacher education programs, as suggested in the previous section of this paper. In addition to the above limitations, it should be borne in mind that this is the first research in the world that has important theoretical and practical significance when it comes to understanding the relationship between capacity for mentalizing and burnout syndrome in a teacher sample.

## Conclusions

Capacity for mentalizing and burnout in teachers are interrelated phenomena. With a good capacity for mentalizing, emotional exhaustion is reduced, as well as burnout in teachers. However, hypermentalizing in teachers as a poor capacity for mentalizing, as it involves less personal investment in communication at work about intentional mental states, is associated with lower burnout. The question is how much the hypomentalizing and hypermentalizing approach of teachers harms the performance of teaching work. Knowledge and skills that enable a good capacity for mentalizing should be included in educational and teacher training programs.

## Supporting information

S1 Data (SAV)
